# Specific Heat and Transport Functions of Water

**DOI:** 10.3390/ijms21020622

**Published:** 2020-01-17

**Authors:** Francesco Mallamace, Carmelo Corsaro, Domenico Mallamace, Enza Fazio, Sow-Hsin Chen, Antonio Cupane

**Affiliations:** 1 Department of Nuclear Science and Engineering, Massachusetts Institute of Technology, Cambridge, MA 02139, USA; sowhsin@mit.edu; 2 Istituto dei Sistemi Complessi (ISC)—CNR, 00185 Rome, Italy; 3 Dipartimento di Scienze Matematiche e Informatiche, Scienze Fisiche e Scienze della Terra (MIFT), Università di Messina, 98166 Messina, Italy; ccorsaro@unime.it (C.C.); mallamaced@unime.it (D.M.); enfazio@unime.it (E.F.); 4 Dipartimento di Fisica e Chimica, Università di Palermo, 90128 Palermo, Italy; antonio.cupane@unipa.it

**Keywords:** water, phase transition, specific heat, diffusivity

## Abstract

Numerous water characteristics are essentially ascribed to its peculiarity to form strong hydrogen bonds that become progressively more stable on decreasing the temperature. However, the structural and dynamical implications of the molecular rearrangement are still subject of debate and intense studies. In this work, we observe that the thermodynamic characteristics of liquid water are strictly connected to its dynamic characteristics. In particular, we compare the thermal behaviour of the isobaric specific heat of water, measured in different confinement conditions at atmospheric pressure (and evaluated by means of theoretical studies) with its configurational contribution obtained from the values of the measured self-diffusion coefficient through the use of the Adam–Gibbs approach. Our results confirm the existence of a maximum in the specific heat of water at about 225 K and indicate that especially at low temperature the configurational contributions to the entropy are dominant.

## 1. Introduction

Water certainly is, in science and technology, of great interest in many research fields, going from chemical physics to life sciences, medicine, biology, agriculture, and engineering [1]. However, despite the large number of studies, its basic properties (as far as those of related systems) are still far from being completely understood. In fact, it has, in contrast with normal liquids, specific behaviors. The first observation is the density maximum ($\rho_{M}$ at 277 K), intuited about four centuries ago (1612) by Galileo Galilei [2] and later discovered in Florence (1667) [3]. As nowadays it is well known, many of these characteristics belong to water in the metastable supercooled state and characterize important thermodynamic response functions like: the expansivity ($\alpha_{P}=-{\left(\partial \ln\rho /\partial T\right)}_{P}$), the compressibilities (isothermal ($\kappa_{T}={\left(\partial \ln\rho /\partial \ln P\right)}_{T}$) and adiabatic ($\kappa_{S}\;=\;{\left(\partial \ln\rho /\partial \ln P\right)}_{S}$)) and the isobaric specific heat ($C_{P}=T{\left(\partial S/\partial T\right)}_{P}$). All of them are associated to microscopic fluctuations: $\kappa_{T}$ with those of the volume *V* or density ($\kappa_{T}=\langle {\left(\delta V\right)}^{2}\rangle /k_{B}TV$), $C_{P}$ with those of the entropy *S* ($C_{P}=\langle {\left(\delta S\right)}^{2}\rangle /k_{B}T$), whereas $\alpha_{P}$ reflects the volume–entropy cross-correlations ($\alpha_{P}=\langle \delta S\delta V\rangle /k_{B}TV$).

Liquid water is considered a prototype of supercooled liquids; i.e., of materials that can be cooled inside a metastable liquid state below their melting temperature ($T_{m}$) down to the homogeneous nucleation temperature ($T_{h}$), and are pressure dependent. It is just in this metastable state that these water response functions fluctuations manifest themselves clearly, and their behaviors give rise to two important thermodynamical realities [4]. One deals with the δS and δV difference on cooling between a normal liquid and water: in the first case, both these fluctuations become smaller as *T* decreases (towards and inside the supercooled phase), but in water, they become more pronounced. Moreover, while in the case of normal liquids they are positively correlated (an increases in δV is accompanied by a similar behavior in δS), for water, below $T_{m}$, they are anticorrelated (an increase in *V* brings an entropy decrease). Thus, this highlights that water cooling is accompanied by an increase in its local order, and these anticorrelations become increasingly pronounced for water inside the supercooled state. The second salient water property, easily observed at ambient pressure, is that its thermodynamic response functions have a diverging (critical) behavior [5]. Extrapolated from their measured values from moderate supercooling down to the lowest temperatures, all these functions appear to diverge at a singular temperature $T_{S}∼225$ K. These results have been related to a possible first-order phase transition between two liquids of the same substance [6,7,8,9,10]; however, it must be stressed that such an idea of a liquid water polymorphism has been fully confirmed in a recent MD study [11].

The Speedy and Angell observations coming from the response functions [5] (on the entropy decreases and the diverging behavior) are at the basis of the water complexity and the enormous interest on it. They can be considered as the starting points of modern research on water leading to the discovery of the central role played by the onset of a water networking with a tetrahedral symmetry driven by the hydrogen bonding (HB) interactions. Nowadays, the corresponding criticality (inside the supercooled regime) is far to be experimentally proven [12]. This despite the incredibly large number of experiments and computational studies that have been made over the years [13].

An improvement in water knowledge is due to its polyamorphism discovery; the existence, in the P−T phase diagram, of two glassy forms with different structure and densities: the high density and the low density amorphous, HDA and LDA respectively [14,15,16,17]. HDA can be formed by LDA, and viceversa; heating LDA at ambient *P*, it undergoes at about 130 K a glass to liquid transition into a highly viscous fluid and then, at $T_{x}=150$ K, it crystallizes into cubic ice (whereas at the same pressure, $T_{h}=235$ K). In the region between $T_{x}$ and $T_{h}$, water cannot stably exist in its bulk liquid form. The HDA glass can also be obtained by pressure-induced amorphization of hexagonal ice ($I_{h}$) contained in emulsion droplets (radius 1–10 μm). Such small volumes suppress the transformation of ice $I_{h}$ in other ice forms upon compression (isothermal) [18]. The HDA has a structure very similar to that of the high-*P* liquid water, suggesting that it is the glass form of high-*P* liquid water, just as LDA may be a glass form of low-*P* liquid water [19,20].

Water polyamorphism and the relative transitions drive the discovery of the proposed existence of two distinct liquid phases characterized by different densities, coexisting in water, namely, the low density (LDL) and the high density (HDL) liquids. Such an idea is supported by the following experimental evidence: (i) high-*T* bulk liquid water, if rapidly cooled at ambient pressure, becomes LDA without crystallization, so that LDA appears directly related to the liquid [21]; (ii) the HDA is connected with the liquid at high *P*, by the ice crystal melting increasing pressure (the two amorphous having small entropies can be considered smoothly connected with the liquid state). Just by taking into consideration the discontinuous LDA–HDA transitions, and the findings of an MD simulation, it has been proposed that liquid supercooled water is really polymorphic, with the two glass phases as their evolution at the dynamical arrest [22,23]. Such a liquid polymorphism can give the explanation for the water’s response functional characteristics in the supercooled state by means of a possible liquid–liquid transition (LLT) and a liquid–liquid critical point (LLCP). The same laboratory has also suggested an alternative interpretation: the singularity-free (SF) scenario which is independent of the polymorphism [24]. However, these works can be considered as a milestone because they brought the interest of the scientific community to the fascinating research subject, that is still an open question: does water have a second critical point? The LLCP also focuses the role of the so-called Widom line (WL), that is the locus of the maximum correlation length. Along this line, the response functions reach extremes and increase on approaching the critical point (the WL can be also considered an extension of the coexistence curve) [25]. Before starting our considerations, we have to mention that the liquid water polymorphism has been definitively proven [26].

The intermolecular water interactions are regulated by the HB: a noncovalent attractive interaction, i.e., an electropositive hydrogen atom on one molecule and an electronegative oxygen atom on another molecule (i.e., the O:H non-covalent van der Waals bond (≃0.1 eV binding energy BE)). In contrast to the HB, there is also a repulsive intermolecular interaction (the Coulomb repulsion between electron lone-pairs on adjacent oxygen atoms), with two H–O covalent bonds sharing the electron lone pairs, ≃4.0 eV. Whereas the HB dominates water in the stable and supercooled regime, the repulsive lone pairs mainly influence the water physics from above the boiling temperature ($T_{b}$) in the sub-critical and critical region (the water critical point CP is located at: $T_{C}=647.1$ K, $P_{C}=22.064$ MPa).

Ordinary ice has a tetrahedral symmetry, in which each water molecule has four nearest neighbors and acts as an H-donor to two of them and an H-acceptor for the other two. Whereas ice is a permanent tetrahedral HB network, the liquid water tetrahedrality is local and transient even if characterized by a lifetime that, on lowering the temperature, strongly increases (more than three orders of magnitude) from values of some picoseconds characteristic of the stable liquid phase ($T>T_{m}$). This is coherent with the response functions behaviors on cooling, the entropy increases (because is $C_{P}>0$) as well as the specific volume ($V_{S}=1/\rho$) due to the progressive increase in the tetrahedral order. In such a way, at ambient pressure, δV and δS can become anticorrelated and $\alpha_{P}<0$. However, the increase in pressure contrasts these ordering effects.

Like the structure, the dynamic or transport water properties are special. When water is sufficiently cold, upon a pressure increase, its diffusivity increases whereas its viscosity decreases; this is due to the *P*-effect that tends to destroy the tetrahedral HB network (and to change the internal structure of a single molecule), by increasing the molecular mobility. The self-diffusion $D_{S}$, decreasing with *T*, has been described by means of scaling laws ($D_{S}\left(T\right)=D_{0}{\left(\left(T-T_{S}\right)/T_{S}\right)}^{-\gamma }$) by proposing [5] a sort of water criticality in the supercooled regime. On the other hand, we must mention that NMR experiments have revealed that there is a crossover temperature at $T^{*}∼315$ K for which, by decreasing *T*, liquid water changes its energetic behavior from Arrhenius to super-Arrhenius [27]. In particular, for T<315 K it is observed that the Stokes–Einstein relation adequately describes the relative temperature dependence of viscosity and diffusion, but above and in the vicinity of such a temperature, the spin lattice relaxation does not follow the viscosity as predicted by this law. Lately, by using the Adam–Gibbs approach (AG) [28], it has been demonstrated that such a temperature can be considered as the onset of the HB tetrahedral network [29]. Then, a combination of NMR and neutron scattering experiments in the deep supercooled regime also showed, by decreasing *T*, another violation of the Stokes–Einstein relation at $T_{L}∼225$ K, accompanied by a dynamical crossover from super-Arrhenius (scaling law) to Arrhenius behavior, thus a Fragile-to-Strong Dynamic Crossover (FSDC) [30]; the FSDC temperature, $T_{L}$, is the locus of the WL.

On the basis of the previous discussions, any exploration regarding bulk water seems forbidden in the region between $T_{x}$ and $T_{h}$ (called no man’s land). Such a constraint can be overcome by using some tricks; one is to confine water in nano-pores (radius 1–2 nm) smaller than nucleation centers [31]; another is to study water inside ice and another one is to melt a multimolecular thickness of ice surface [32]. Just this latter approach, by using the fast pulsed–laser-heating technique, has been able to determine (for temperatures between 180 and 262 K) the crystalline-ice growth rate and, thus, the liquid water self-diffusion under ultrahigh-vacuum conditions [32]. In this case, a film of 25-ML (water mono layer ML, i.e., about 5.5 nm) of amorphous solid water (ASW), deposited on poly-crystalline ice surface (at very low pressure $10^{-5}$ MPa), by means of rapid heating, was transformed into supercooled liquid water so that the crystalline-ice growth rate can be determined and, thus, the liquid-water diffusivity. Hence, this latter scenario is completely different from the geometrical constraints imposed by the confinement.

Although these tricks have allowed discovering many important water properties, like the existence of a density minimum (predicted more than a century ago by Percy W. Bridgman [33]) and the development of proper models for the supercooled state [34], they are the subject of severe criticisms, because some people consider confined water a different physical “variety” respect to the bulk.

The large lot of studies made in confined water, by the use of very different experimental techniques, ranging from the scattering (elastic and inelastic) to the calorimetry including NMR, ESR and FTIR spectroscopy, have given many results on what happens in the temperature region between $T_{x}$ and $T_{h}$ like: a density minimum [35,36], the evidence of the LDL, the existence of the Widom line, a minimum with negative values in the expansivity $\alpha_{P}$, a maximum in the specific heat $C_{P}$ and in the isothermal compressibility $\kappa_{T}$, and the dynamical crossover from fragile to strong glass-former behavior coincident with the WL, the observation at this crossover of the violation of the Stokes–Einstein relation [30] (with the consequent onset of the dynamical heterogeneities and the decoupling between the translational and rotational modes) etc. [31]. It must be stressed that the existence of a maximum in $\kappa_{T}$, just at $T_{L}$, was originally proposed by the analysis of sound velocity data measured in bulk water as a function of the frequency and the wavevector v(ω,q) [37]. A definitive proof of this has been recently given by a proper study of the water structure factor, S(q→0), in micrometer-sized water droplets (and, thus, in bulk) at ambient pressure, by using X-ray scattering (X-ray lasers) [38].

As previously mentioned, these experimental results on water confined in nanopores were subject to criticisms, essentially based on the assumption that the confined water density profile is not uniform across the pore, that a significant amount of water is absorbed outside the pore, and that the dynamic crossover in supercooled confined water could be a crystallization transition in the larger pore or surface water [39,40]. Many of the studies on the water properties (theoretical and experimental) have been (and are) the subject of hot debate. An example on experimental findings regards the recent X-ray experiment on the $\kappa_{T}$ maximum [38], questioned because “the reported data do not lead to clear conclusions about the origins of water’s characteristics” [41]. The arguments used were the following: (i) for an inappropriate use of the Ornstein–Zernike formalism (which is reliable near a critical point) and (ii) for the required knowledge of ρ, not available for T<239.74 K [41]; and lately iii) for the validity of the used temperature calibration scheme [42]. However, the corresponding answer stresses that, together with this compressibility maximum, the density–density correlation length has also been mesured observing for such a function a maximum at 229 K, so providing a direct evidence of the Widom line that is another support to the LLT [43].

In this context, the recent pulsed-laser-heating dynamical data, supporting the hypothesis that the unusual thermodynamics of liquid water are also responsible for its dynamic characteristics, should be considered. Such an experiment not only gives evidence of the dynamical crossover observed in confined water (at about $T_{L}$ ) but, in addition, the corresponding results appear to be consistent with either the LLCP (or the SF) at positive pressures, as both are consistent with a continuous entropic change in no man’s land. In addition, because the current experiments furnish continuous $D_{S}$ values, they provide no evidence for a liquid–liquid transition line extending to negative pressures [44]. It is observed that at the lowest temperatures, the measured diffusion data are considerably larger than predicted [45], a situation due to the observed breakdown of the Stokes–Einstein relation [30].

Among the many possible studies on water properties, the confinement gives us the opportunity to explore transport quantities as far as calorimetric data. The specific heat is just one of the most essential thermodynamic quantities for looking into the hydrogen bonding properties in supercooled water. Our study is addressed just to explore the measured specific heat $C_{P}\left(T\right)$ in bulk and confined water, in a very large temperature range (80<T<350 K) at ambient pressure under different confinements. After that, taking into account the transport parameters (diffusivity), measured under different conditions and, by using the Adam–Gibbs prediction for $D_{S}\left(T\right)$, we evaluate the water configurational entropy ($S_{C}$) and, thus, the configurational specific heat $C_{P}^{C}\left(T\right)$. The Adam–Gibbs approach, the comparison with the available $C_{P}\left(T\right)$ data and the results of recent theoretical MD studies, support the hypothesis that the unusual thermodynamics of liquid water are also responsible for its dynamic characteristics [45,46,47]. Moreover, there is a consistence with either the singularity-free scenario or the liquid–liquid critical point at positive pressures or with a continuous change in entropy across the no man’s land.

## 2. Methods, Results and Discussion

### 2.1. Methods

**Figure 1:**
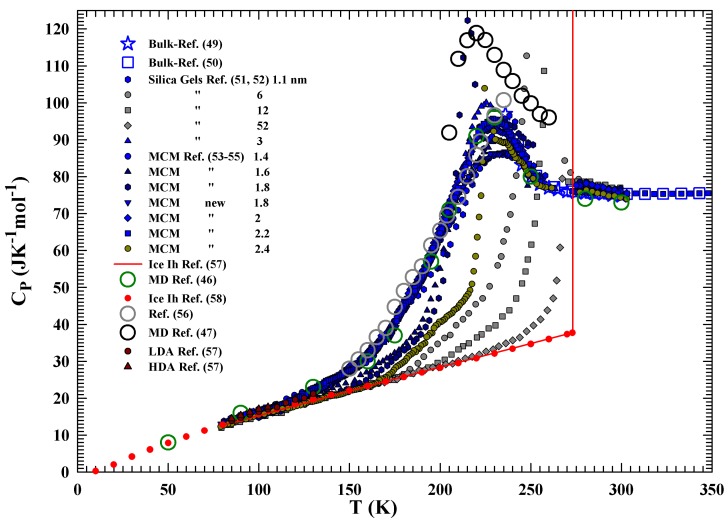
The $C_{P}\left(T\right)$ data measured, at the ambient pressure, in bulk [49,50], confined water [51,52,53,54,55,56] and those obtained by the two mentioned MD simulation studies [46,47]. Specifically, the bulk water data cover the range 236<T<350 K (thus including the supercooled regime), wheras confined water data are measured under different confining materials with various pores in the range 80<T<300 K. The figure also reports data of ice Ih 10–273 K [57,58], of LDA and HDA [57].

Figure 1 illustrates the water $C_{P}\left(T\right)$ data, experimentally measured and evaluated by means of recent MD simulations at ambient pressure, under different conditions. Specifically, the figure reports data measured in bulk water (in the range 236<T<300 K, thus including the supercooled regime [48,49,50]), confined water [51,52,53,54,55,56], ice Ih [57,58], LDA, HDA [57] and the cited simulation studies with the TIP4P/2005 model potential [46,47]. However, although both the two mentioned MD studies report a maximum in $C_{P}\left(T\right)$ in the same temperature interval (220–230 K), there are some discrepancies about their values that could be associated with the lack of a complete equilibration in the data of ref. [46], especially for the runs at the lowest temperatures (below 170 K). As can be observed, for confined water, some $C_{P}\left(T\right)$ data coming from different experiments and in a very large temperature region (80–300 K, i.e., well inside the no man’s land up to the region of the amorphous ice), are reported. In particular, we report the studies of water confined in: (i) silica gels with pore sizes in the range of 1.1–5.2 nm: [51,52]; (ii) cylindrical MCM nanopores with different sizes in the range of 1.4–4.2 nm [53,54,55]. More precisely, the Figure 1 data deal with silica gels—CARiACT—(1.1, 3, 6, 12, and 52 nm pore sizes) and MCM nanotubes coming from previous [53,54,55] (1.4, 1.6, 1.8 nm) and new experiments (1.8, 2, 2.2 and 2.4 nm). New data were acquired by using the same experimental procedure and conditions reported in ref [55]: the same heating and cooling rate of 12 K/h. The specific heat of water in a silica xerogel matrix (with a rather narrow distribution of pore sizes and an average dimensions of about 2 nm) [56,59], is also reported for comparison. The red line and red data points deal with the $C_{P}\left(T\right)$ of ice Ih [57,58]. The illustrated data come from two different experimental techniques: adiabatic method [51,52] and the usual differential scanning calorimetry (DSC). In particular, the new data were acquired with this last technique (the same previously used for MCM samples, hydrated after a complete drying [55]) working with a Perkin–Elmer DSC 8500. The heat capacity of the water within the pores is derived by subtracting, from the heat capacity of the sample, the contributions of the excess water. The first method has been just developed to account for the specific properties of a glass as it ages, and it can be used to separate the properties of water around the surface from those of the internal one. It is, however, surprising to see the agreement between the $C_{P}\left(T\right)$ data of confined water and those of the MD simulations [46].

The specific heat experiments on glass-forming materials are made in a thermal cycle: first, the sample is cooled starting from the liquid stable phase toward, and below, its estimated glass transition temperature. After that, the sample is heated returning to the starting point. As it is well known, the rate of the cooling and heating phases usually must be the same in order to have reproducible and comparable data. The illustrated data deal with the $C_{P}\left(T\right)$ during the heating phase. The specific heat values in 257–273 K are not reported (could not be derived because of the large fusion contribution which must be subtracted from the observed values).

### 2.2. Results and Discussion

From the reported data, it is evident that in the case of large and relatively large pore sizes, most of the water crystallized as ice on cooling. In the case of little pores, water remains in the liquid state down to the lowest measured temperature, namely, this is the case of silica gels with pore sizes of 1.1 and 3 nm, whereas for MCM this happens for some samples (with pores of 1.4 nm for the previous data [53,54] and of 1.8, 2 and 2.2 nm for the new data). At the same time, a $C_{P}$ maximum at about 230 K, i.e., the water FSDC temperature, can be observed in all these confined water data. It is also observable that the $C_{P}$ maximum values ($C_{P}^{\max}$) increased only in proportion to the pore size (and, thus, with the fraction of the pores internal water), whereas its temperature appears essentially not dependent on that. From this, we have an important suggestion related to the absolute relevance of the HB network on water: the HB formation (and the related LDL phase) progresses in nanopore water and is essentially the same as that of bulk water. Strong hydrogen bonds are formed on cooling by arranging the neighboring water molecules at the tetrahedral positions, but in contrast with the very stable ice structure, the LDL network has a dynamical character (the HB living time starting from the pico-second (in the stable phase) increases with continuity by decreasing *T* [32,34]).

All these results on the water specific heat, inside no man’s land, together with the self diffusion data behaviors (in bulk and confined water [27,32,34]) and a very recent study just on the water density (at different *T* and *P* [60]), propose that the observed $C_{P}\left(T\right)$ behavior (Figure 1) may be essentially due to the HB network properties and their thermodynamical aspects, more specifically to the configurational contribution to the state functions (entalpy and entropy). This was originally proposed in terms of theoretical considerations linked to the LLCP hypothesis [45].

The connections of the system dynamics to the thermal energy, entropy and $C_{P}\left(T\right)$ changes, are, as it is well known, of primary significance in the study of supercooled liquids and of their dynamical arrest, and for their understanding, many different approaches have been used. Nowadays, the energy landscape is considered a good approach: how a system’s thermodynamical state point fluctuates in its possible potential energy configurations, depending on the geometry of the molecular arrangement, particularly with reference to structural relaxations and transport properties [61,62,63].

**Figure 2:**
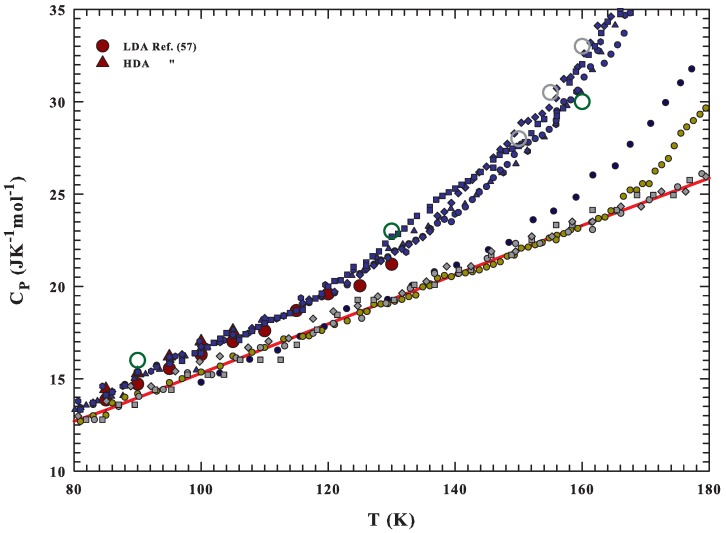
The $C_{P}$ data of Figure 1 reported in an enlarged temperature scale (80–180 K). The values of LDA and HDA are reported as dark red circles and triangles, respectively [57].

In the solid phase, as far as in the glass one, the vibrational motions are the primary contributions to $C_{P}$. Instead, in the liquid phase, the configurational component, due to the change in the number arrangement with *T* that the structure of the liquid explores, is the dominant one, plus a minor vibrational component (caused by a force constant and frequency changes with *T*). Hence, the glass phase has most of the vibrational contribution, and on heating above $T_{g}$, $C_{P}$ begins to gain the configurational contribution in a *T*-dependent manner regulated by the system structure and interactions. All the $C_{P}$ evolutions previously illustrated represent such a situation, and to have a clear vision of such a physical picture, Figure 2 reports the $C_{P}$ data in an enlarged temperature scale (80–180 K), highlighting as dark red circles and triangles the values of the LDA and HDA, respectively [57]. The confirmation that essentially the vibrational modes contribute to $C_{P}\left(T\right)$ in the glass region, can ascertained by observing the data of this latter figure and on considering that the densities of these two amorphous phases are, at 90 K and at the ambient pressure, very different: $\rho_{LDA}≃0.94$, and $\rho_{HDA}≃1.167$ g/cm${}^{3}$ [60], whereas their $C_{P}$ values are very close ($C_{P}^{LDA}≃14.7$ and $C_{P}^{HDA}≃15.3$ JK${}^{-1}$mol${}^{-1}$), and with a little difference respect to the ice Ih ($C_{P}^{Ih}≃14$ JK${}^{-1}$mol${}^{-1}$).

**Figure 3:**
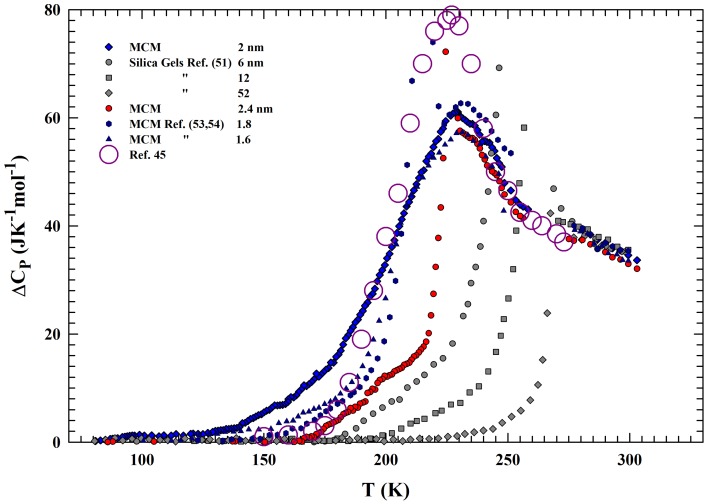
The difference between the measured specific heat of water and ice Ih. According to ref. [45], such a quantity gives a good estimation of the configurational contribution: $C_{P}^{Conf}=\Delta C_{P}=C_{P}-C_{P}^{Ih}$. The data deal with water in the following samples: silica gels (6, 12, and 52 nm pore sizes), MCM (1.6, 1.8 nm [53,54] and 2, 2.4 nm (new data)). The values coming from thermodynamical considerations are also reported [45].

On this basis, we can assume that according to ref. [45], the difference between the measured specific heat of water and of ice Ih, can give a good estimation of the configurational contribution: $C_{P}^{Conf}=\Delta C_{P}=C_{P}-C_{P}^{Ih}$. Figure 3 reports such quantity for water in the following samples: silica gels (6, 12, and 52 nm pore sizes), MCM (1.6, 1.8 nm [53,54] and 2, 2.4 nm (new data)). Unlike the MCM of 2 nm in which confined water always remains in the liquid state for all the experiment, in the other samples, water freezes during cooling. By heating, these latter samples show a different evolution toward the melting, just depending on the fraction of water remaining in the liquid state on cooling with respect to the crystallized one (bigger for larger pores). From the low *T* data evolution before the melting, such a fraction can be easily estimated, and ranges from ∼50% (for the smallest pore sizes 1.6 nm) to ∼8% (12 nm); whereas for the sample with the widest size (52 nm), all water has frozen. It can be noticed that the melting temperatures are: ∼210 K for samples with pore sizes 1.6, 1.8 nm; ∼224 K for that of 2.4 nm and ∼248, ∼260 and ∼270 K for the remaining silica gels with, 6, 12 and 52 nm pore size, respectively. The figure also reports, as red open circles, the $C_{P}^{Conf}$ evaluation for bulk water, made according to some thermodynamical considerations and the $C_{P}$ knowledge of ice, and liquid water outside the range $T_{x}-T_{h}$ [45]. It must be mentioned that such a study is the first, to our knowledge, proposing a $C_{P}$ maximum for liquid water inside the no man’s land (at ∼225 K). However, a comparison with the experimental data shows an agreement with the maximum temperature and with similar *T*-evolution; the only difference is at the lowest *T*, below 200 K (presumably due to the $C_{P}$ underestimation at 150 K, ≈2 JK${}^{-1}$mol${}^{-1}$). For completeness, it must be said that in such a study, the water self diffusion $D_{S}$ behavior has been predicted starting from the assumption of a thermodynamical connection between supercooled water and LDA. The water thermodynamical functions (entropy, specific heat and enthalpy), at 150 and 236 K, have been used to estimate the excess entropy, $S_{ex}\left(T\right)$, and the configurational one at intermediate temperatures, and then by means of the AG theory to calculate $D_{S}\left(T\right)$. In fact, the same authors confirm a proportionality between $S_{conf}$ and $S_{ex}$ [45].

**Figure 4:**
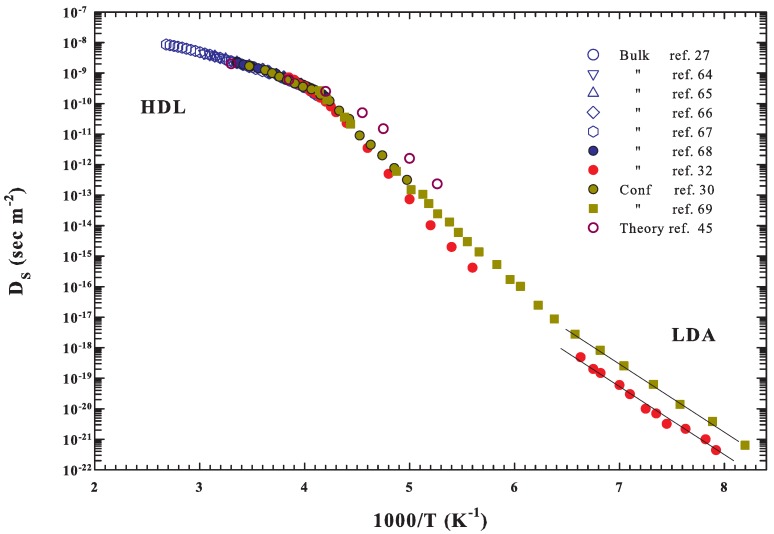
A collection of self-diffusion data is reported in an Arhhenius representation, $D_{S}$ vs. 1000/T. Data come from bulk water (as blue symbols [27,64,65,66,67,68]), fused amorphous water (dark red [32]) together with MCM confined water (NMR [30] and dielectric relaxation data [69], dark yellow); data coming from the theoretical study, obtained from the evaluated $C_{P}^{Conf}$ by using the Adam–Gibbs (dark pink open circles), are also reported [45]. These data cover a very large *T*- range from 373 to 120 K, with an overall variation of about 12 orders of magnitude, without the presence of any singularities. The two straight lines indicate the strong behavior at the lowest temperatures.

The reported data give us a confirmation that for samples with pore sizes that are less than 2.4 nm, water remains liquid through the whole of no man’s land to well inside the amorphous region, and the evaluated $C_{P}$ evolves with continuity with a well defined maximum, which is a possible sign of the LLCP. Such a situation holds also for the silica gels of 3 nm. Hence, the observed continuity seems to propose that measured $C_{P}$ is the same at all the explored temperatures, for both confined and bulk water. A confirmation on this can be obtained from the measured transport functions, in particular, from the self-diffusion $D_{S}$ data, by means of the entropy, in conjunction with the theory of Adam and Gibbs [28]. At the end, this theory allows the calculation of the configurational $C_{P}^{Conf}$ from the measured $D_{S}$. Now, experimental data are available in all the regions of the reported $C_{P}$ experiments. There are data from water in MCM samples and those from the fast pulsed-laser-heating technique, on multi-layered amorphous water (5.5 nm) under vacuum fused by the light pulse, that can be considered as bulk water [32]. The collection of these data is reported in Figure 4 in an Arrhenius representation. The figure shows the $D_{S}$ data vs 1000/T coming from bulk water (as blue symbols [27,64,65,66,67,68]), fused amorphous water (dark red [32]) and MCM confined water (NMR [30] and dielectric relaxation data [69], dark yellow); data coming from the cited theoretical studies, obtained from the evaluated $C_{P}^{Conf}$ by using the Adam and Gibbs (dark pink [45] open circles), are also reported. Note that these values are calculated from simulations of the SPC/E model.

The experimental data in Figure 4 cover a very large temperature range from 373 to 120 K, with an overall $D_{S}$ variation of about 12 orders of magnitude without the presence of singularities. However, although inside the supercooled regime there is a difference of about an order of magnitude in $D_{S}$, the Arrhenius behavior of confined and “bulk” data is essentially the same. From these data, a fragile-to-strong transition (or dynamic crossover) just at ∼ 225 K (1000/T=4.444 K${}^{-1}$) and a second crossover at ∼ 187 K (1000/T=5.348 K${}^{-1}$) to a lower activation energy, are observable as predicted [70]. Hence, the water configurational entropies are obtained from these $D_{S}$ data according to the AG prediction equation:

(1) $$D_{S}=D_{S0}\exp\left(\frac{A}{TS_{Conf}}\right)$$

where *A* and $D_{S0}$ are constant. A data fitting in the range of bulk water (373–237 K) gives $D_{S0}\;=\;1.07$ 10${}^{-7}$ m${}^{2}$sec${}^{-1}$ and A=−31.75 kJmol${}^{-1}$; values in good agreement with that of ref. [45].

**Figure 5:**
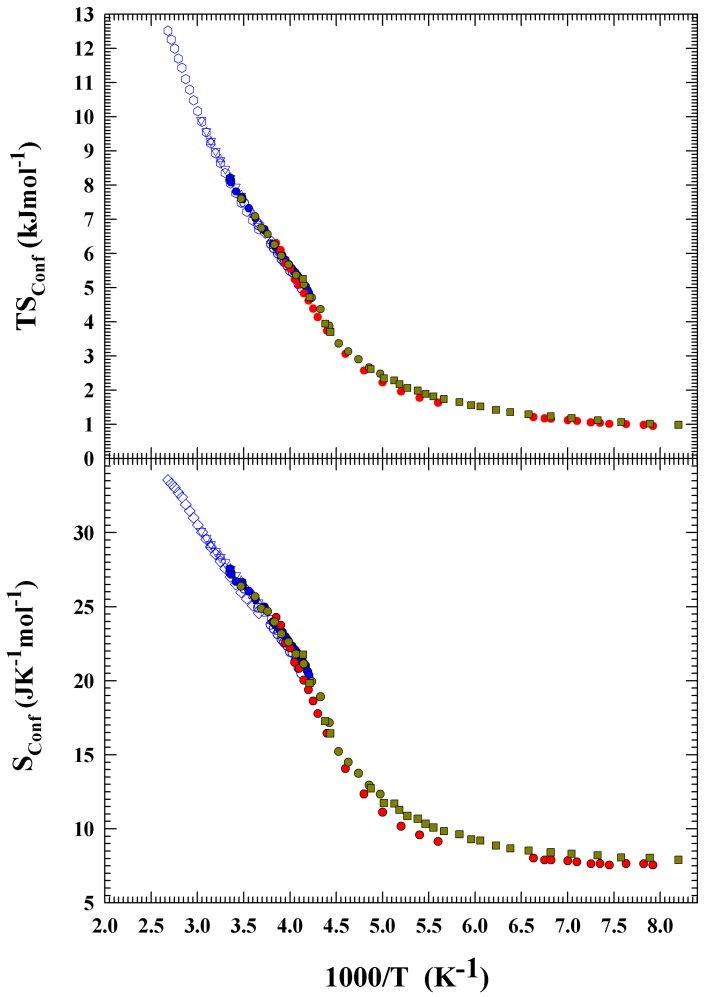
The $TS_{Conf}$ and $S_{Conf}$ obtained from the transport data acording to the AG equation, in the top and bottom panels, respectively.

The $TS_{Conf}$ and $S_{Conf}$ results so obtained are proposed in Figure 5, in the top and bottom panels, respectively. From the data behavior, a slope change in both panels can be observed at about 315 K (1000/T=3.18 K${}^{-1}$), whereas at about 225 K (1000/T=4.444 K${}^{-1}$) there is a flex point. Finally, after a $S_{Conf}$ data smoothing, the temperature derivative is made and $C_{P}^{Conf}$ is calculated in all the temperature range. The MCM and fast pulsed-laser-heating technique data are treated separately. Despite the difference in the $D_{S}$ data inside the deep supercooled regime, the difference in the same *T* region between $TS_{Conf}$ and $S_{Conf}$ is a few percentage points.

**Figure 6:**
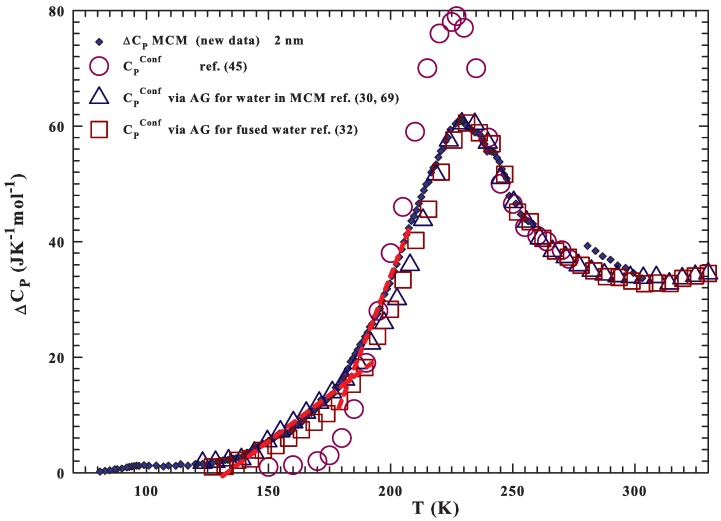
The configurational $C_{P}^{Conf}=T{\left(\partial S_{Conf}/\partial T\right)}_{P}$ obtained from the transport data compared with those mearasured as $\Delta C_{P}$ in confined liquid and evaluated by using thermodynamical concepts [45].

The obtained data of $C_{P}^{Conf}=T{\left(\partial S_{Conf}/\partial T\right)}_{P}$ are illustrated in Figure 6 as large symbols; the data of the mentioned theoretical study [45] are also reported. The difference between the current data and these latter is due to the fact that, at low temperatures, the experimental self diffusion is, as reported in Figure 4, considerably smaller than the one theoretically predicted.

As it can be observed, the data behaviors are characterized, as expected, by a well defined maximum in $C_{P}^{Conf}$ and also from an overlap of the values evaluated by the self diffusion coefficients and experimentally measured in liquid-confined water as $\Delta C_{P}=C_{P}-C_{P}^{Ih}$. We recall that the fragile-to-strong crossover, coincident with the WL and violation of the Stokes–Einstein relation, is located just near the temperature of the water $C_{P}\left(T\right)$ maximum, also observed in MD simulations [25] and NMR experiments [71]. From the structural point of view, the fragile-to-strong crossover, as far as the water $C_{P}\left(T\right)$ maximum, are strongly related with the local order (or the average number of HB’s, nHB) [72]. As it is well known, nHB, which is less than four at high temperatures (when water is essentially HDL), approaches four at low temperature; whereas the metastable supercooled regime is characterized by the HB tetrahedral network (the LDL phase). The fractional weight of these two different liquid forms reaches 50% just at the crossover temperature [72]. A manifestation of a fully coordinated network is the appearance of anomalous suppression of long-range density fluctuations in correspondence with the glass transition [73].

The $D_{S}\left(T\right)$ behavior suggests a second crossover (strong-to-strong) at 180 K observable in the $C_{P}^{Conf}\left(T\right)$ (see e.g., the two dashed red lines for T<200 K), which again might be associated with changes in the hydrogen-bonding network of liquid water. The difference between the apparent activation energy for *T*≶ 180 K may be due to a change in the molecular mobility in a transition from a “defective” liquid (T>180 K) to a “fully” hydrogen-bonded liquid (i.e., with a fixed number of bonds) for T<180 K, characterized by a smaller activation energy. It must be also observed that a further decrease in the temperature will lead first at the highly viscous fluid phase and after to the LDA.

## 3. Conclusions

We conclude by observing that the present experimental data, about specific heat in confined water and self-diffusion in confined and bulk stages, explain in a quantitative way that water is a liquid in which there is a striking change in physical properties as the temperature is changed between the melting point and the glassy state regime. The explored data cover, at ambient pressure, a very large temperature interval from the stable liquid phase, from near that of boiling to the amorphous phase (LDA).

All the observed thermodynamical properties are connected to the fact that water molecules aggregate through forming the tetrahedral hydrogen bond network. This is consistent with the theoretical observations of the cooperative effect of the HB network [74] and of the corresponding ordering involving an LDL-like environment [47]. It is therefore suggested that the involved energies and functions are correlated with the average number of hydrogen bonds and the thermal effects on the molecular rearrangements. The main suggestion is, thus, that the local order and its configurations drive all the unusual water properties on decreasing temperature up to the dynamical arrest. Therefore, we have used the entropy-based Adam–Gibbs theory, developed to describe the relaxation of liquids approaching their glass transitions, providing the temperature variations of the self-diffusion constant $D_{S}\left(T\right)$.

In this frame, we have related the measured specific heat $C_{P}\left(T\right)$ (present study and literature data) in bulk and confined water to the transport data just to highlight the relevant effect of local configurational order. For more precision, we studied how the changes in the inter- and intra-molecular water structural properties, as far as their correlations, determine its intriguing thermodynamics. To do this, we benefited from (i) recent experimental data on $D_{S}\left(T\right)$, which in practice explore bulk liquid water also inside no man’s land and (ii) the measured specific heat of water confined in pores which diameter (<2.2 nm) avoids crystallization, thus allowing the measurement of such an important thermodynamical function in the same large T-range of $D_{S}\left(T\right)$. The only assumption made, as previously said, is that the excess entropy defined as $S_{ex}\equiv S_{liquid}-S_{crystal}$ can be considered as the configurational one ($S_{conf}\equiv S_{liquid}-S_{vib}$); i.e., the mechanism responsible for the liquid to glass transitions is essentially a rearrangement process changing the position and orientation of each molecule in liquids. In other words, we have assumed that $S_{vib}=S_{crystal}$, an invalid situation in principle for simple liquids although widely used. In the case of water, in temperature regions where the effects of the HB network and the consequent reorientation of molecules within such an HB quasi-lattice are dominant, all the contributions to the entropy (beyond those of the crystal) are to be considered configurational. The results illustrated in Figure 6 seem to confirm such a point of view.

## Abbreviations

The following abbreviations are used in this manuscript:

AG	Adam–Gibbs
HB	Hydrogen Bond
LDL	Low Density Liquid
HDL	High Density Liquid

## References

[1] Ball P. (2001). Life’s Matrix: A Biography of Water.

[2] Galilei G. (1612). Intorno Alle Cose, Che Stanno in sù L’acqua, ò Che in Quella si Muovono. Discorso al Serenissimo Don Cosimo II Gran Duca di Toscana.

[3] Magalotti L. (1667). Saggi di Naturali Esperienze Fatte Nell’Accademia del Cimento Sotto la Protezione del Serenissimo Principe Leopoldo di Toscana e Descritte dal Segretario di essa Accademia.

[4] Debenedetti P.G., Stanley H.E. (2003). Supercooled and Glassy Water. Phys. Today.

[5] Speedy R.J., Angell C.A. (1976). Isothermal compressibility of supercooled water and evidence for a thermodynamic singularity at −45 °C. J. Chem. Phys..

[6] Rapoport E. (1967). Model for Melting-Curve Maxima at High Pressure. J. Chem. Phys..

[7] Némethy G., Scheraga H.A. (1962). Structure of Water and Hydrophobic Bonding in Proteins. I. A Model for the Thermodynamic Properties of Liquid Water. J. Chem. Phys..

[8] Davis C.M., Litovitz T.A. (1965). Two-State Theory of the Structure of Water. J. Chem. Phys..

[9] Jhon M.S., Grosh J., Ree T., Eyring H. (1966). Significant-Structure Theory Applied to Water and Heavy Water. J. Chem. Phys..

[10] Kamb B. (1965). Structure of Ice VI. Science.

[11] Palmer J., Martelli F., Liu Y., Car R., Panagiotopoulos A., Debenedetti P. (2014). Metastable liquid–liquid transition in a molecular model of water. Nature.

[12] Pettersson L., Henchman R., Nilsson A. (2016). Special issue on: Water—The Most Anomalous Liquid. Chem. Rev..

[13] Palmer J., Poole P.H., Sciortino F., Debenedetti P. (2018). Advances in Computational Studies of the Liquid-Liquid Transition in Water and Water-Like Models. Chem. Rev..

[14] Mishima O., Calvert L., Whalley E. (1984). Melting ice I at 77 K and 10 kbar: A new method of making amorphous solids. Nature.

[15] Mishima O., Calvert L., Whalley E. (1985). An apparently first-order transition between two amorphous phases of ice induced by pressure. Nature.

[16] Mishima O. (1996). Relationship between melting and amorphization of ice. Nature.

[17] Burton E.F., Oliver W.F., McLennan J.C. (1935). The crystal structure of ice at low temperatures. Proc. R. Soc. Lon. Ser. A Math. Phys. Sci..

[18] Loerting T., Salzmann C., Kohl I., Mayer E., Hallbrucker A. (2001). A second distinct structural “state” of high-density amorphous ice at 77 K and 1 bar. Phys. Chem. Chem. Phys..

[19] Amann-Winkel K., Gainaru C., Handle P.H., Seidl M., Nelson H., Böhmer R., Loerting T. (2013). Water’s second glass transition. Proc. Natl. Acad. Sci. USA.

[20] Amann-Winkel K., Böhmer R., Fujara F., Gainaru C., Geil B., Loerting T. (2016). Colloquium: Water’s controversial glass transitions. Rev. Mod. Phys..

[21] Bruggeller P., Mayer E. (1980). Complete vitrification in pure liquid water and dilute aqueous solutions. Nature.

[22] Poole P., Sciortino F., Essmann U., Stanley H.E. (1992). Phase behaviour of metastable water. Nature.

[23] Santra B., Distasio R.A., Martelli F., Car R. (2015). Local structure analysis in ab initio liquid water. Mol. Phys..

[24] Sastry S., Debenedetti P.G., Sciortino F., Stanley H.E. (1996). Singularity-free interpretation of the thermodynamics of supercooled water. Phys. Rev. E.

[25] Xu L., Kumar P., Buldyrev S.V., Chen S.-H., Poole P.H., Sciortino F., Stanley H.E. (2005). Relation between the Widom line and the dynamic crossover in systems with a liquid–liquid phase transition. Proc. Natl. Acad. Sci. USA.

[26] Soper A.K., Ricci M.A. (2000). Structures of High-Density and Low-Density Water. Phys. Rev. Lett..

[27] Simpson J.H., Carr H.Y. (1958). Diffusion and Nuclear Spin Relaxation in Water. Phys. Rev..

[28] Adam G., Gibbs J.H. (1965). On the Temperature Dependence of Cooperative Relaxation Properties in Glass-Forming Liquids. J. Chem. Phys..

[29] Mallamace F., Corsaro C., Mallamace D., Vasi C., Stanley H.E. (2013). The thermodynamical response functions and the origin of the anomalous behavior of liquid water. Faraday Discuss..

[30] Chen S.-H., Mallamace F., Mou C.Y., Broccio M., Corsaro C., Faraone A., Liu L. (2006). The violation of the Stokes–Einstein relation in supercooled water. Proc. Natl. Acad. Sci. USA.

[31] Mallamace F., Baglioni P., Corsaro C., Spooren J., Stanley H.E., Chen S.-H. (2011). Transport properties of supercooled confined water. Rivista del Nuovo Cimento.

[32] Xu Y., Petrik N.G., Smith R.S., Kay B.D., Kimmel G.A. (2016). Growth rate of crystalline ice and the diffusivity of supercooled water from 126 to 262 K. Proc. Natl. Acad. Sci. USA.

[33] Bridgman P. (1912). Water, in the Liquid and Five Solid Forms, under Pressure. Proc. Am. Acad. Art. Sci..

[34] Cerveny S., Mallamace F., Swenson J., Vogel M., Xu L. (2016). Confined Water as Model of Supercooled Water. Chem. Rev..

[35] Mallamace F., Branca C., Broccio M., Corsaro C., Mou C.Y., Chen S.-H. (2007). The anomalous behavior of the density of water in the range 30 K < T < 373 K. Proc. Natl. Acad. Sci. USA.

[36] Erko M., Wallacher D., Hoell A., Hauß T., Zizak I., Paris O. (2012). Density minimum of confined water at low temperatures: A combined study by small-angle scattering of X-rays and neutrons. Phys. Chem. Chem. Phys..

[37] Mallamace F., Corsaro C., Stanley H.E. (2013). Possible relation of water structural relaxation to water characteristics. Proc. Natl. Acad. Sci. USA.

[38] Kim K.H., Späh A., Pathak H., Perakis F., Mariedahl D., Amann-Winkel K., Sellberg J.A., Lee J.H., Kim S., Park J. (2017). Maxima in the thermodynamic response and correlation functions of deeply supercooled water. Science.

[39] Soper A.K. (2013). Radical re-appraisal of water structure in hydrophilic confinement. Chem. Phys. Lett..

[40] Soper A.K. (2013). Density profile of water confined in cylindrical pores in MCM-41 silica. J. Phys. Cond. Matters.

[41] Caupin F., Holten V., Qiu C., Guillerm E., Wilke M., Frenz M., Teixeira J., Soper A.K. (2018). Comment on “Maxima in the thermodynamic response and correlation functions of deeply supercooled water”. Science.

[42] Goy C., Potenza M.A.C., Dedera S., Tomut M., Guillerm E., Kalinin A., Voss K.O., Schottelius A., Petridis N., Prosvetov A. (2018). Shrinking of Rapidly Evaporating Water Microdroplets Reveals their Extreme Supercooling. Phys. Rev. Lett..

[43] Kim K.H., Späh A., Pathak H., Perakis F., Mariedahl D., Amann-Winkel K., Sellberg J.A., Lee J.H., Kim S., Park J. (2018). Response to Comment on “Maxima in the thermodynamic response and correlation functions of deeply supercooled water”. Science.

[44] Pallares G., El Mekki Azouzi M., Gonzalez M.A., Aragones J.L., Abascal J.L.F., Valeriani C., Caupin F. (2014). Anomalies in bulk supercooled water at negative pressure. Proc. Natl. Acad. Sci. USA.

[45] Starr F., Angell C.A., Stanley H. (2003). Prediction of entropy and dynamic properties of water below the homogeneous nucleation temperature. Phys. A Stat. Mech. Appl..

[46] Saito S., Bagchi B. (2019). Thermodynamic picture of vitrification of water through complex specific heat and entropy: A journey through “no man’s land”. J. Chem. Phys..

[47] Martelli F. (2019). Unravelling the contribution of local structures to the characteristics of water: The synergistic action of several factors. J. Chem. Phys..

[48] Angell C.A., Sichina W.J., Oguni M. (1982). Heat capacity of water at extremes of supercooling and superheating. J. Chem. Phys..

[49] Tombari E., Ferrari C., Salvetti G. (1999). Heat capacity anomaly in a large sample of supercooled water. Chem. Phys. Lett..

[50] Archer D.G., Carter R.W. (2000). Thermodynamic Properties of the NaCl + H_2_O System. 4. Heat Capacities of H_2_O and NaCl(aq) in Cold-Stable and Supercooled States. J. Phys. Chem. B.

[51] Oguni M., Maruyama S., Wakabayashi K., Nagoe A. (2007). Glass Transitions of Ordinary and Heavy Water within Silica-Gel Nanopores. Chem. Asian J..

[52] Oguni M., Kanke Y., Namba S. (2008). Thermal Properties of the Water Confined within Nanopores of Silica MCM-41. AIP Conf. Proc..

[53] Nagoe A., Kanke Y., Oguni M., Namba S. (2010). Findings of Cp Maximum at 233 K for the Water within Silica Nanopores and Very Weak Dependence of the Tmax on the Pore Size. J. Phys. Chem. B.

[54] Oguni M., Kanke Y., Nagoe A., Namba S. (2011). Calorimetric Study of Water’s Glass Transition in Nanoscale Confinement, Suggesting a Value of 210 K for Bulk Water. J. Phys. Chem. B.

[55] Tombari E., Salvetti G., Johari G.P. (2012). Specific Heat and Transformations of Water in 1.4 and 1.8 nm Pore-MCMs. J. Phys. Chem. C.

[56] Cupane A., Fomina M., Piazza I., Peters J., Schirò G. (2014). Experimental Evidence for a Liquid-Liquid Crossover in Deeply Cooled Confined Water. Phys. Rev. Lett..

[57] Handa Y.P., Mishima O., Whalley E. (1986). High-density amorphous ice. III. Thermal properties. J. Chem. Phys..

[58] Wagner W., Riethmann T., Feistel R., Harvey A. (2011). New Equations for the Sublimation Pressure and Melting Pressure of H_2_O Ice Ih. J. Phys. Chem. Ref. Data.

[59] De Michele V., Levantino M., Cupane A. (2019). Hysteresis in the temperature dependence of the IR bending vibration of deeply cooled confined water. J. Chem. Phys..

[60] Mallamace F., Corsaro C., Mallamace D., Fazio E., Chen S.-H. (2019). Some considerations on the water polymorphism and the liquid–liquid transition by the density behavior in the liquid phase. J. Chem. Phys..

[61] Stillinger F.H. (1988). Supercooled liquids, glass transitions, and the Kauzmann paradox. J. Chem. Phys..

[62] Mallamace F., Branca C., Corsaro C., Leone N., Spooren J., Chen S.-H., Stanley H.E. (2010). Transport properties of glass-forming liquids suggest that dynamic crossover temperature is as important as the glass transition temperature. Proc. Natl. Acad. Sci. USA.

[63] Yip S., Short M.P. (2013). Multiscale materials modelling at the mesoscale. Nat. Mater..

[64] Gillen K.T., Douglass D.C., Hoch M.J.R. (1972). Self-Diffusion in Liquid Water to −31 °C. J. Chem. Phys..

[65] Holz M., Heil S.R., Sacco A. (2000). Temperature-dependent self-diffusion coefficients of water and six selected molecular liquids for calibration in accurate 1H NMR PFG measurements. Phys. Chem. Chem. Phys..

[66] Mills R. (1973). Self-diffusion in normal and heavy water in the range 1-45.deg. J. Phys. Chem..

[67] Price W.S., Ide H., Arata Y. (1999). Self-Diffusion of Supercooled Water to 238 K Using PGSE NMR Diffusion Measurements. J. Phys. Chem. A.

[68] Prielmeier F.X., Lang E.W., Speedy R.J., Lüdemann H.D. (1988). The Pressure Dependence of Self Diffusion in Supercooled Light and Heavy Water. Berichte der Bunsengesellschaft für Physikalische Chemie.

[69] Sjöström J., Swenson J., Bergman R., Kittaka S. (2008). Investigating hydration dependence of dynamics of confined water: Monolayer, hydration water and Maxwell–Wagner processes. J. Chem. Phys..

[70] Ito K., Moynihan C., Angell C.A. (1998). Thermodynamic determination of fragility in liquids and a fragile-to-strong liquid transition in water. Nature.

[71] Mallamace F., Corsaro C., Broccio M., Branca C., Gonzalez-Segredo N., Spooren J., Chen S.-H., Stanley H.E. (2008). NMR evidence of a sharp change in a measure of local order in deeply supercooled confined water. Proc. Natl. Acad. Sci. USA.

[72] Mallamace F., Broccio M., Corsaro C., Faraone A., Majolino D., Venuti V., Liu L., Mou C.Y., Chen S.-H. (2007). Evidence of the existence of the low-density liquid phase in supercooled, confined water. Proc. Natl. Acad. Sci. USA.

[73] Martelli F., Torquato S., Giovambattista N., Car R. (2017). Large-Scale Structure and Hyperuniformity of Amorphous Ices. Phys. Rev. Lett..

[74] Stokely K., Mazza M.G., Stanley H.E., Franzese G. (2010). Effect of hydrogen bond cooperativity on the behavior of water. Proc. Natl. Acad. Sci. USA.

